# Association of genes with phenotype in autism spectrum disorder

**DOI:** 10.18632/aging.102473

**Published:** 2019-11-19

**Authors:** Sabah Nisar, Sheema Hashem, Ajaz A. Bhat, Najeeb Syed, Santosh Yadav, Muhammad Waqar Azeem, Shahab Uddin, Puneet Bagga, Ravinder Reddy, Mohammad Haris

**Affiliations:** 1 Research Branch, Sidra Medicine, Doha, Qatar; 2 Department of Psychiatry, Sidra Medicine, Doha, Qatar; 3 Weill Cornell Medicine, Doha, Qatar; 4 Translational Research Institute, Hamad Medical Corporation, Doha, Qatar; 5 Center for Magnetic Resonance and Optical Imaging, Department of Radiology, Perelman School of Medicine at The University of Pennsylvania, Philadelphia, PA 19104, USA; 6 Laboratory Animal Research Center, Qatar University, Doha, Qatar

**Keywords:** signaling pathways, genetic variants, neuronal activity, synaptic plasticity, gene transcription

## Abstract

Autism spectrum disorder (ASD) is a genetic heterogeneous neurodevelopmental disorder that is characterized by impairments in social interaction and speech development and is accompanied by stereotypical behaviors such as body rocking, hand flapping, spinning objects, sniffing and restricted behaviors. The considerable significance of the genetics associated with autism has led to the identification of many risk genes for ASD used for the probing of ASD specificity and shared cognitive features over the past few decades. Identification of ASD risk genes helps to unravel various genetic variants and signaling pathways which are involved in ASD. This review highlights the role of ASD risk genes in gene transcription and translation regulation processes, as well as neuronal activity modulation, synaptic plasticity, disrupted key biological signaling pathways, and the novel candidate genes that play a significant role in the pathophysiology of ASD. The current emphasis on autism spectrum disorders has generated new opportunities in the field of neuroscience, and further advancements in the identification of different biomarkers, risk genes, and genetic pathways can help in the early diagnosis and development of new clinical and pharmacological treatments for ASD.

## INTRODUCTION

**Figure 1:**
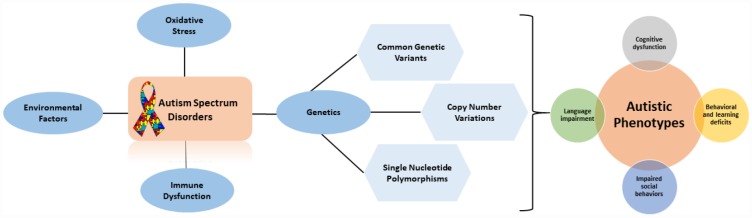
**Flow diagram outlining the factors that contribute to autistic phenotypes.**

Autism spectrum disorder (ASD) is a complex neurological disorder that affects an individual’s development by impairing social interaction and communication and causes stereotypical behaviors that disrupt the anatomy and functional connectivity in the brain. Most common psychiatric comorbidities found to be associated with autism include anxiety and intellectual disability. Individuals with autism have impaired speech [1, 2] and tend to have limited social interaction mostly due to their own limitation of social skills and due to their failure to understand self-inner mental states [3]. The impairment of speech in affected individuals depends on the severity of the autism disorder as autistic individuals tend to repeat certain words or phrases they hear others say, their speech might sound more formal and they exhibit repetitive behaviors [4]. The prevalence of autism is on the rise and the global prevalence of ASD has been reported to be 1 in 160 persons, according to the World Health Organization (WHO) (2014). Based on a parent survey, the recent prevalence of ASD in the U.S. is reported to be 1 in 45 children [5]. A study conducted in 2006 in the United Kingdom reported an ASD prevalence of 38.9/10,000 in 9 to 10-year-olds [6], while another study conducted by the National Autistic Society (2014) reported that 1/100 children are affected with ASD. In Gulf Cooperation Council (GCC) countries, the prevalence of ASD was reported to range from 1.4–29 in 10,000 individuals [7]. The rise in the prevalence of ASDs is a result of many contributing factors. Some researchers believe in the biome depletion theory, which states that an overreaction of maternal immune response is an underlying factor responsible for the development of ASD in children. In addition, as our immune systems co-evolve with many types of pathogens, a lack of these pathogens within urban and developed areas can cause over-reactivity of the immune system [8]. Other factors include increased exposure to environmental toxins that can damage the genetic structure of an individual, thereby increasing genetic susceptibility (Figure 1) [9–11]. A study by Velasquez-Manoff (2012) reported that autoimmune disorders and immune dysregulation in pregnancy are also found to contribute to the rise of ASD [12]. The genetics involved in autism are of considerable importance as they help us identify various genes, proteins, and signaling pathways found in ASD. The study of genes and genetic changes found in patients with ASD can help unravel the genetic architecture underlying ASD and can aid in early diagnosis and clinical treatment. In the present study, the genomic changes that occur in ASD with different genetic variations will be discussed, and the role of ASD risk genes in gene transcription and translation regulation processes, neuronal activity modulation, synaptic plasticity, signaling pathways, and the novel candidate genes that play a significant role in the pathophysiology of ASD will be examined.

## Genetic basis of autism

Many genes associated with ASD are found in circadian entrainment, which indicates a heterogeneous genetic etiology for ASD [13]. Some rare mutations cause the development of syndromic autism. For example, about 30% of patients with Fragile-X Syndrome (FXS) have also been found to develop ASD [14]. A subgroup of patients with Cortical Dysplasia-focal Epilepsy (CDFE), Rett Syndrome and Tuberous Sclerosis Syndrome have also found to express autistic behavior [15, 16]. Copy number variations (CNVs) that involve the deletion or duplication of loci are also responsible for syndromic autism. For example, mutations in SHANK3/PROSAP2 that are found in Phelan-McDermid syndrome are associated with ASD [17]. Mutations in different genes, such as STXBP1, KCNQ4, MYH14, GJB6, COL11A1, UBE3A, KATNAL2, and THRA, have also been associated with ASD [13].

## Common genetic variants

Three independent genome-wide association studies (GWAS) have reported genetic variants commonly associated with autism. Out of these 3 studies, 2 have used 0.5 million single nucleotide polymorphisms (SNPs) and have discovered that they are linked at 5p14.1 [18] and 5p15.2 [19] loci. Similarly, in a different study, other associations for rs4141463 have been found at loci 20p12.1. by using one million SNPs [20]. Another ASD risk gene, CNTNAP2, has been found to have a common genetic SNP variation caused by an alteration of functional connectivity in the frontal lobes [21]. A high contribution of these common genetic variants has been associated to autism liability, estimated to be about 40% in simplex and 60% in multiplex families. The contribution of these common variants is of great significance for better diagnosis in autism but the SNPs associated with ASD are still under research [22].

## Copy number variations (CNVs)

CNVs are found to be a source of autism risk. A study conducted on autism-affected families reported excess gene duplications and deletions in affected autistic individuals compared to the normal controls [23]. Rare *de novo* and inherited events found in pathogenic CNVs involved genes associated with autism, such as CHD2 [24–26], HDAC4, and GDI1, SETD5, HDAC9, and MIR137 [23]. CNVs were found to be highly penetrant in females with autism and in individuals with X syndrome protein targets. It was also found that *de novo* CNV-affected genes converge on neuronal signaling and networks associated with the functioning of synapse and regulation of chromatin [23].

In an ASD gene study, 6 risk loci, namely 1q21.1, 3q29, 7q11.23, 16p11.2, 15q11.2-13, and 22q11.2, associated with autism disorders were reported by analyzing *de novo* CNVs that were tested within 2,591 families. The study found out that genes within small *de novo* mutations tend to overlap with high risk genes associated with ASD [27]. Most of the affected individuals were found to carry a *de novo* causative mutation, as well as deleterious mutations [28]. Gene disrupting mutations, such as frame-shift, splice site, and nonsense mutations, were most frequently found in individuals with ASD [28]. Three percent of the autistic individuals were found to have gene disrupting mutations that were present on both maternal and paternal chromosomes, and 2% of autistic males had a 1.5 fold increase in complete loss of function mutations for X-chromosomes, compared to males without ASD [29].

## Gene aberrations associated with ASD

A study associated with the identification of novel candidate genes in ASD-associated pathways revealed several deletions and gene disruptions in many ASD cases, wherein eighteen deletions were detected at the 3p26.3, 4q12, 14q23, and 2q22.1 regions [30]. Candidate genes associated with GABAergic signaling and neural development pathways were revealed by the evidence provided by case specific CNVs. These genes include a GABA type A receptor associated protein (GABARAPL1), a postsynaptic GABA transporter protein (SLC6A11), and a GABA receptor allosteric binder known as diazepam binding inhibitor (DBI). A genetic overlap between ASD and other neurodevelopmental disorders was also reported, including genes such as GRID1, GRIK2, and GRIK4, which include glutamate receptors, NRXN3, SLC6A8, and SYN3, and are responsible for synaptic regulation. These CNVs are associated with ASD heritability and can help to uncover new etiological mechanisms underlying ASD [30].

## Genetic variation in ASD

There is a substantial variation in the ASD genetic architecture and the heterogeneity of ASD is due to the genetic variability that underlies this disorder. A single mutation is enough to cause ASD and several thousand low-risk alleles can contribute to the development of ASD [31]. There are many rare variants that can contribute to the risk of developing ASD and there is extreme locus heterogeneity in ASD due to copy-number variant data and mutations involving the alteration of proteins [32]. Many of the ASD genes share a common pathway that affects neuronal and synaptic homeostasis. For example, social impairment and speech problems in ASD individuals are due to a single copy mutation SHANK3 [33]. This shows that many of the ASD associated genes are part of a large number of molecular pathways or mechanisms that are related to other neuropsychiatric conditions [34].

## Novel candidates in ASD

Many mutations have been reported in CHD8, an ATP-dependent chromodomain helicase responsible for the regulation of CTNNB1 [35] and p53 pathway [36]. CHD8 has been investigated in many exome studies and is considered as a novel candidate for ASD [37–39]. In addition, the SCN2A gene, which encodes a voltage-gated sodium channel, plays an important role in the generation of action potentials in neurons. These mutations are most frequently found in Identity Disorder (ID), with some cases also showing signs of autism [40, 41]. In autism probands, three truncating mutations of GRIN2B and SYNGAP1 and TBR1 associated with dual-specificity tyrosine phosphorylation-regulated kinase 1A (DYRK1A) play an important role in excitatory signaling in ASD. While, GRIN2B is associated with learning and memory, and targeted sequencing has linked it with various neurodevelopmental disorders, including ASD [38].

An ASD study showed the differential expression of autism candidate genes in lymphoid cell lines of autistic individuals [42]. The study reported the upregulation and increased expression of protein argininosuccinate synthetase (ASS), which acts as an important brain signaling molecule and is involved in controlling the rate-limiting step in the production of nitric oxide (NO), is associated with ASD [42]. An upregulation in the mRNA expression levels of ITGA2B encoding glycoprotein αIIβ was found in ASD affected individuals. This elevated level of expression can disrupt the cellular morphology in affected individuals [43]. Another autism study that identified abnormal brain gene expression patterns in autistic brains via whole-genome analysis of mRNA levels and CNVs reported that in young autistic individuals, the highly dysregulated pathway was the adenosine A2A receptor-signaling pathway [44]. Adenosine receptors play an important role in the development and functioning of the brain, as well as the synaptic plasticity, motor, and cognitive function and neuronal stem cell proliferation regulation [44]. Many studies have reported the association between Purkinje cells (PCs) and ASD [45–47]. Additionally, many studies have reported a significant reduction in the number of PCs in the post-mortem brains of patients who were affected with ASD [48–51]. Lower expression of neurotrophins NT3 and NT4, which play an important role in the climbing fiber system development in PCs, was also reported in affected autistic individuals [52]. Downregulation of neural and muscle specific alternative splicing regulator (A2BP1/FOX1) [53] and the MOCOS gene was also reported in ASD individuals [54]. Language and speech disorders in ASD were found to be result of mutations in the FOXP2 and CNTNAP2 genes [55]. The FOXP1 gene, which is a transcription repressor and is important for normal brain development and functioning, was found to be elevated in individuals with ASD. This indicates the involvement of FOXP1, FOXP2, and CNTNAP2 genes in the pathogenesis of ASD [56]. Synaptic components such as Nav1.2 channel, which is a voltage-gated ion channel involved in action potential propagation, neuronal pacemaking and Cav1.3 channel, which is an excitability-transcription coupling were found to be mutated in autistic patients [57]. Another ASD study showed the presence of an additional copy of 22q13/SHANK3 in a boy with Asperger syndrome that caused severe social communication impairment [58]. The SHANK3 gene is involved in the enlargement of dendritic spine heads as evidenced in mice [59]. There have also been reports of X-linked mutations in NLGN3 and NLGN4 in ASD that affect synapse formation and maintenance, which is important for speech development and social communication [58]. An ASD study that identified rare variants in mGLUR signaling pathway reported rare and deleterious variants in the SHANK3, TSC1, and TSC2 genes in non-syndromic autism individuals [60]. HOMER1, which is an autism risk gene, is considered an important component of the postsynaptic density (PSD) proteins network. This network creates a link between gene products associated with autism and neuroligins [60]. Disruption of mGluR5-Homer1 interactions can cause the development of phenotypes associated with autism [61]. Mutations in SYNGAP1, which is involved in the negative regulation of the Ras/ERK pathway and synaptic transmission, have also been identified in ASD [60]. The UBE3A and GABA receptor genes, which are expressed in the central nervous system, are located at the15q11-13 locus, and are also associated with autism [62]. O’Roak, Vives, Fu, et al. (2012) also discovered that six genes (GRIN2B, TBR1, CHD8, PTEN, TBL1XR1, and DYRK1A) contribute to 1% of ASDs and were found to be sporadic. Parikshak et al. [63] found that genes associated with autism tightly bind together in modules that are responsible for human cortical development and biological functions, including transcriptional regulation and the development of synapses.

Disruption of the NRXN1 gene in autism reported that in autistic individuals, the amino acid alterations in the NRXN1 gene are not frequently present as compared to non-autistic individuals in an NRXN1 gene coding sequence scan [64]. Two missense changes seen in the residues of the leader sequence of a-NRXN1 and epidermal growth factor (EGF)-like domain suggests these changes in NRXN1 might be a contributing factor in developing autism [64].

## Autistic phenotypes

To identify the genes affected by rare *de novo* CNVs, in autism, a network-based analysis of genetic associations (NETBAG) is used [65]. The network forming genes are associated with autism and are involved in the development of synapse, neuron motility and axon targeting. In addition to the WNT signaling pathway, which is responsible for neural circuits formation and dendrite morphogenesis regulation, there is a reelin signaling pathway that plays a significant role in neuron motility and autistic phenotypes [66, 67]. This network also includes deleted in colorectal carcinoma (DCC) protein, which is responsible for guiding the axon in autism disorder [68]. In addition, other proteins involved in the regulation of actin network that are used in axonal morphogenesis, such as p21-activated kinase (PAK) and LIM-domain containing protein kinase (LIMK) and any malfunction in these proteins influences autistic phenotypes as they play a significant role in dendrite/axon signaling [65]. Autistic individuals are also found to have increased density of spine in portions of cerebral cortex with over-connectivity in local brain regions [69, 70]. This indicates that malfunctioning of the neuronal and synaptic connectivity is central to autism, and several genes could be related to postsynaptic density and actin remodeling that could contribute to autistic phenotypes [65]. Also, higher ASD rates have been reported in individuals with low copy repeats LCR-A to B region deletion compared to individuals without deletions. This might contribute to autistic phenotypes in disorders related to 22q11.2 (also known as DiGeorge syndrome) in addition to decreased adaptive functioning [71].

**Figure 2:**
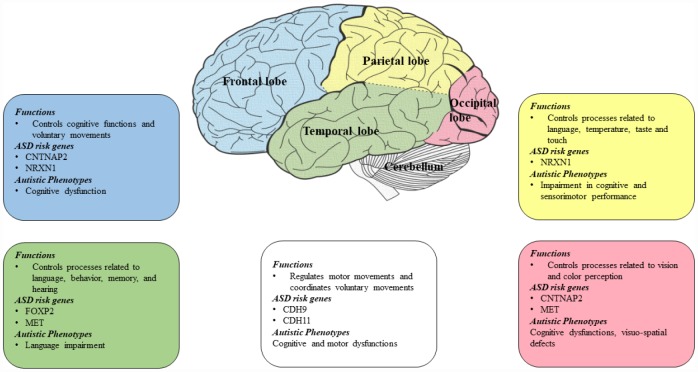
**Diagram showing ASD risk genes and autistic phenotypes associated with different lobes of the brain.** CNTNAP2 is found to be associated with frontal and occipital lobes of the brain [70, 73]. NRXN1 is found to be linked with parietal and frontal lobes [74]. FOXP2 is found to be linked with temporal lobe and MET in occipital and temporal lobes [75]. Cadherins (CDH9 and CDH11) are found to be linked with the cerebellum region [37].

There are ASD risk genes that contribute to autistic phenotypes and are associated with different lobes of the brain (Figure 2). The frontal lobe is the largest and controls different cognitive functions such as memory, behaviors, language and voluntary movements. The parietal lobe controls sensorimotor planning, language and touch. The temporal lobe is mainly involved in controlling the semantic and recognition memory and the occipital lobe, which is the smallest lobe in the brain mainly controls visual processes [72]. One of the most important ASD risk gene is CNTNAP2 and it is found to be associated with frontal and occipital lobes of the brain [70, 73]. While NRXN1 is found to be linked with parietal and frontal lobes [74]. FOXP2 is found to be linked with temporal lobes and MET in occipital and temporal lobes [75]. Genetic findings have associated cadherins as an ASD risk gene [18, 76–78] and a recent study identified the expression of cadherins CDH9 and CDH11 in the ASD-relevant areas of the cerebellum in mice and reported high expression of these genes in segregated populations of the Purkinje cells present in the cerebellum [79].

## Gene regulation in ASD

### DNA methylation

A global methylation profiling study in lymphoblastoid cell lines derived from autistic monozygotic twins and their non-autistic siblings revealed decreased expression of RORA and BCL-2 proteins in autistic individuals as compared to the controls [80]. The study confirms how DNA methylation in idiopathic autism affects the epigenetic regulation of gene expression and the molecular changes associated with brain pathobiology in ASD [80].

### Postsynaptic translational regulation

Postsynaptic density plays a critical role in the neural transmission and maturation of synapsis and forms the basis of the etiology of ASD [81]. The encoded proteins of autism-associated genes located in the postsynaptic density are associated with FMRP, and there is a localization of mutated protein found in Fragile-X syndrome, which is responsible for the synthesis of proteins at the postsynaptic density [82]. The SHANK2 and SHANK3 genes are found in the postsynaptic density, which bind to neuroligins and are involved in the glutamatergic response in ASD, as well as in language and social cognition development [83]. Other candidate genes of autism include NF1, PTEN, MET, TSC1, TSC2, and CYFIP1, that are located in the duplication region (15q11–13) [84–87]. Mutations in ASD genes also implicate the protein metabolism at the synapse that is modulated by the ubiquitination pathways [88]. For example, UBE3A, which is the Angelman syndrome gene, has an important role in this pathway, in addition to genes such as FBXO40, RFWD2, USP7, and PARK2 [89, 90]. This indicates that remodeling and maintenance of the synapse functioning is an important determinant in the pathology of ASD [91].

### Modulation of neuronal activity

Mutations in neurexin and neuroligin families are associated with the pathophysiology of ASD [92]. Together, neurexins and neuroligins are involved in the modulation of excitatory and inhibitory synaptic functions [93]. The genes of these super families that play a significant role in ASD include NRXN1, NLGN1, NLGN3, CNTN4, CNTN6, and CNTNAP2 [94]. Neuronal activity is influenced by genes such as GRIN2B, SCN1A, and SCN2A, and they are involved in the mediation of synaptic plasticity. In addition, they encode for ion channels [32]. Neuronal activity that regulates transcription factors also regulates other genes, including UBE3A, PCDH10, DIA1, and NHE9/SLC9A9 [95, 96]. Imbalances in excitation and inhibition in brain regions have been found in knockout ASD mouse models of genes, such as NRXN1, SHANK3, FMR1, CNTNAP2, and these knockout models had social interaction impairments and reduced ultrasonic vocalizations that overlapped behavioral endophenotypes relevant to ASD [91].

### Synaptic plasticity

Synaptic plasticity is affected by cytogenetic abnormalities, such as the duplication of maternal allele 15q11–q13 and genetic syndromes like Rett or Fragile-X syndrome associated with ASD. In idiopathic autism, most commonly identified synaptic gene mutations include NLGN4X [96, 98] or SHANK3 [33] and NLGN3. Abnormalities in the formation of synapse and disrupted pathways, such as GTPase/Ras signaling and neurogenesis, are revealed by the analysis of genes affected by rare CNVs [99, 100]. The identification of specific ASD genes are found to be resisted by some *de novo* or inherited CNVs, for example, the 16p11 region, recur at the same locus in individuals who are not related [101]. This indicates a locus and allelic heterogeneity in ASD [101]. Another X-linked gene GLRA2 deletion has been identified in autism disorder [102]. This gene encodes the glycine receptors (GlyR) α2 subunits. These glycine receptors are involved in the mediation of inhibitory neurotransmission in the nervous system. Mutations in GLRA2 results in synaptic plasticity, language delay, cognitive and social impairments, as well as altered glycinergic signaling [102]. Mutations in Calcium voltage-gated channel subunit alpha1 C (CACNA1C) might contribute to NMDA-receptor independent synaptic plasticity associated with ASD [103]. A study showed that mutations in CACNA1C cause alterations in calcium homeostasis that contribute to the development of ASD [104].

## Genes and brain connectivity

**Figure 3:**
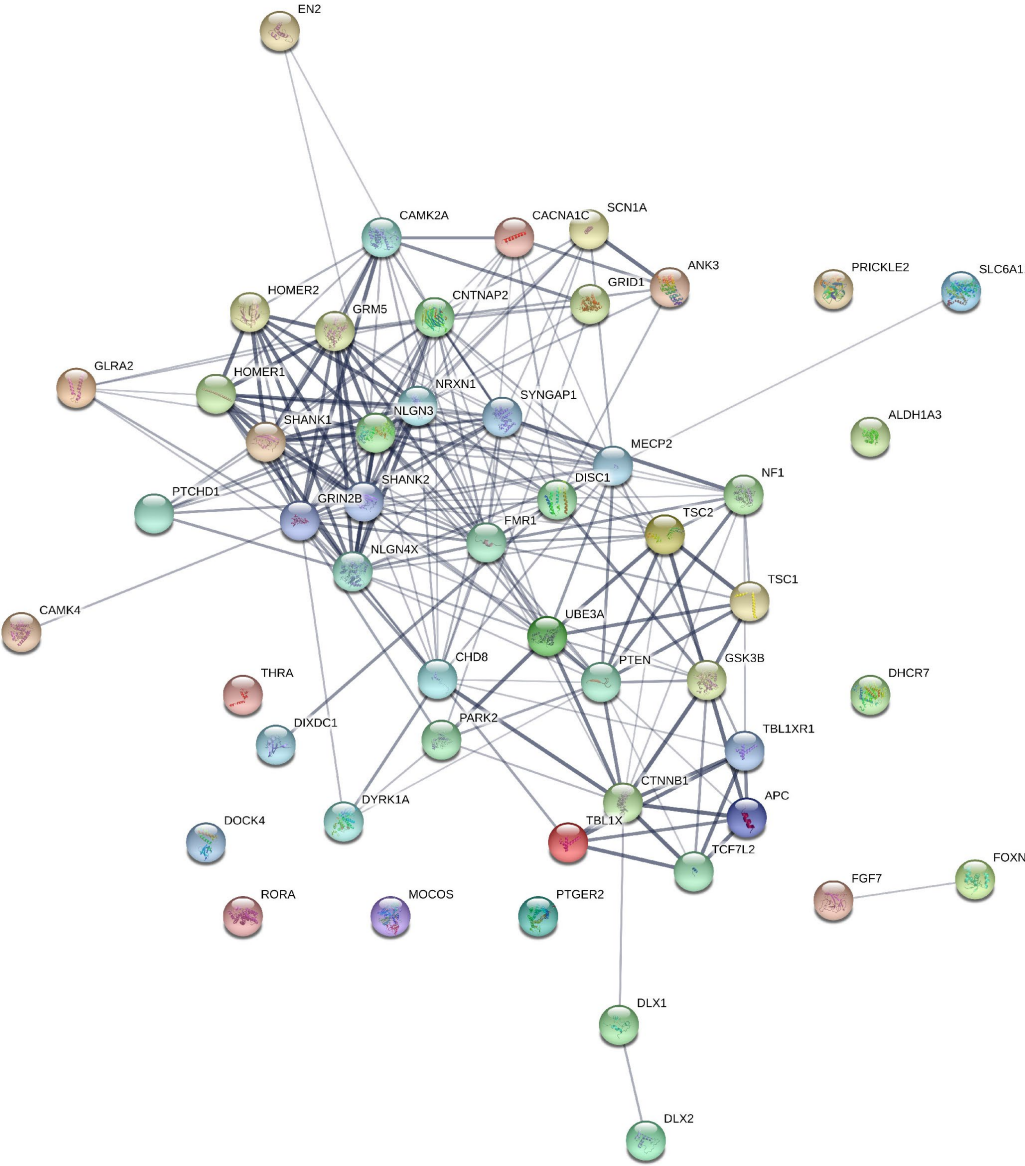
**Gene interaction map for ASD genes generated using string1 webserver.** Thickness of the line indicates the strength of the interaction between the genes. All sources are used to generate the interaction model with default medium confidence interaction score of 0.4. Ref: https://string-db.org/.

Genes involved in ASD are related to each other within many processes, including neuronal and synaptic development, modulation, protein synthesis, calcium signaling, oxytocin pathways, mTOR, and various transcriptional mechanisms (Figure 3) [105]. ASD potential endophenotypes include brain connectivity and morphology alterations [106]. A study by Frazier et al. (2014) on ASD subjects carrying heterogenous germline PTEN mutations showed cognitive malfunctioning and abnormalities in the white matter with reductions in PTEN protein compared to the healthy controls [117]. Alterations were also found in the gene-brain pathway based on the rs1858830 MET risk allele by differences seen in activation and deactivation patterns of fMRI in response to social stimuli, as well as the structural and functional connectivity in the temporal-parietal region of the brain in ASD subjects [108].

## Genome-wide association study (GWAS)

Many GWASs have been performed in ASD [89, 109–111]. Within the intergenic region between CDH9 and CDH10 cell adhesion genes, a linkage disequilibrium block has been reported [18]. Another gene called Semaphorin 5A (SEMA5A), which plays an important role in axonal guidance and the development of neurons is found to be a susceptibility gene for ASD as a study has found a *de novo* microdeletion of SEMA5A in ASD and ID patients [112]. There was another genome-wide significance reported in the macro domain containing 2 (MACROD2) gene at an intronic SNP [113]. Replication and identification of a common variation on chromosome 5p14.1 associated with autism was reported by another GWAS study [57, 114].

## Wnt-signaling in ASD

**Figure 4:**
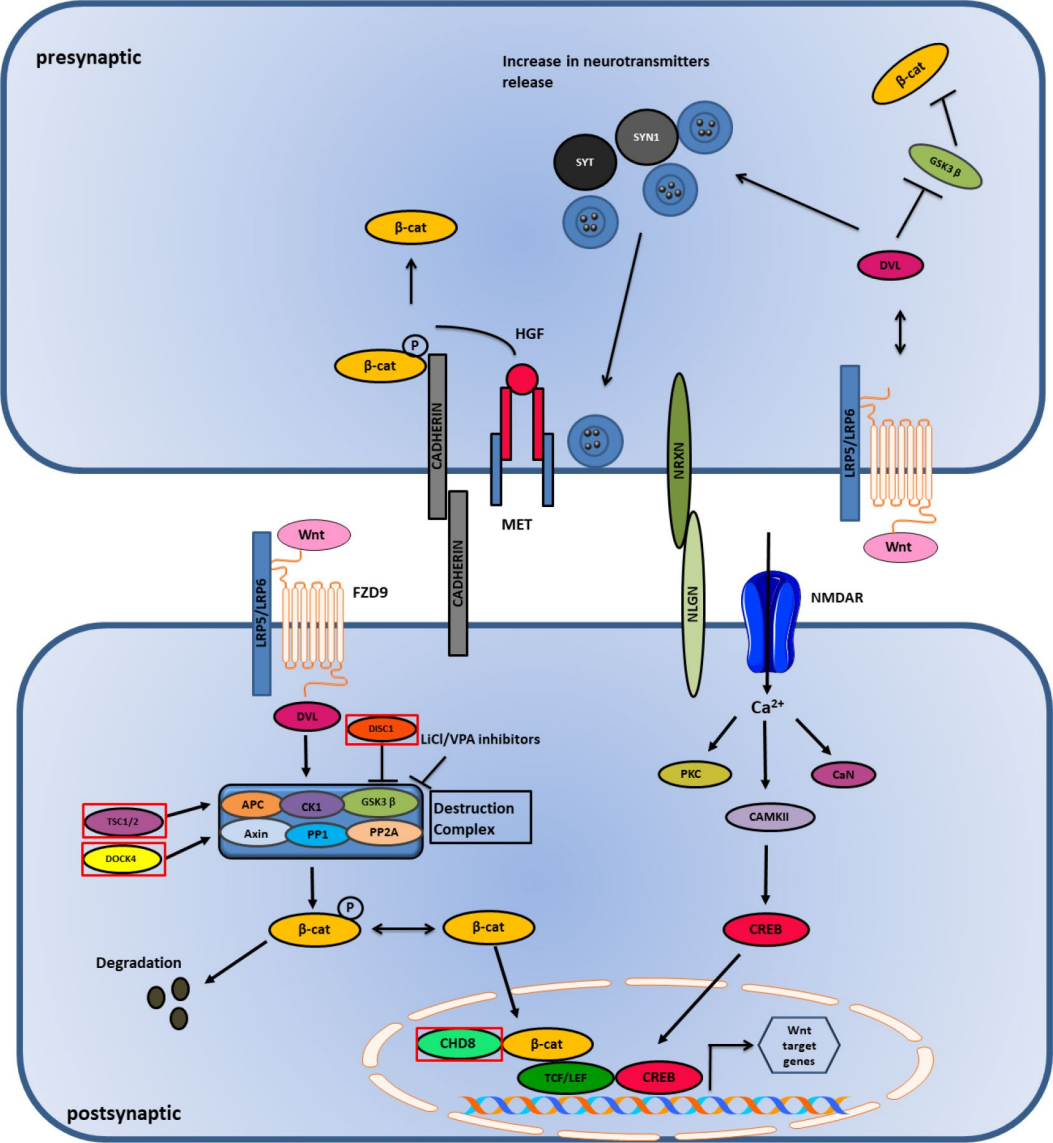
**Wnt and Ca2+ pathway in ASD.** Wnt binds to LRP5/LRP6 receptor and stabilizes β-catenin in the nucleus and cytoplasm. High influx of Ca2+ ions causes activation of CAMK and CREB genes which initiates transcription in the postsynaptic side. Genes mutated in ASD are shown in red boxes.

Wnt-signaling plays an important role in the differentiation and morphology of neurons, and neurotransmission [115]. This pathway is found to be dysregulated in individuals affected with ASD [116]. Dysregulation of this pathway is found to affect the cortical and spine patterning and morphology with cytoarchitecture disruption in the cortex of the affected brains of autistic individuals [117, 118]. Glycogen Synthase Kinase-3 (GSK3), which is a Wnt-signaling pathway component, plays an important role in ASD development as it was found to be hyperactive and caused impaired social interaction and increased anxiety in a GSK3 knockout mouse model [119]. Another gene, known as CHD8, which regulates the Wnt signaling pathway and promotes transcriptional factor activity in the brain, is found to be associated with ASD [120]. Knockout of CHD8 gene during cortical development resulted in the downregulation of the CHD8 gene, which lead to the reduction of the TCF/LEF transcription factor family, causing a defect in the development of the brain [121] (Figure 4). This proves that the dysregulation of the Wnt-signaling pathway affects the cortical patterning and synaptic development in individuals with ASD [122].

The DISC1 gene is associated with Wnt signaling and ASD and is involved in the inhibition of GSK3β that leads to the activation of β-catenin; thus, it acts as a positive regulator of the Wnt/β-catenin signaling [123]. Mutations in DISC1 fail to inhibit GSK3β and cause suppression of the Wnt/β-catenin signaling pathway [124]. Mutations in an intracellular Wnt/β-catenin signal pathway protein, DIX domain containing-1 (DIXDC1), displayed impairment in social behavior, coupled with anxiety and depression in mice models [125] and also showed reduction in the dendritic spines and glutamatergic synapses in brains of the experimental mice. It has been suggested that the functional sequence variants of DIXDC1 can manifest as behavioral syndromes in human due to its restricted tissue distribution property in the late developmental and postnatal central nervous system [126]. The TSC1 and TSC2 genes are also found to play a significant role in Wnt signaling [127]. These genes are associated with GSK3β and Axin and help in the degradation of β-catenin leading to the inhibition of Wnt/β-catenin mediated gene transcription. Mutations in these genes are found to increase Wnt signaling, while overexpression of these genes caused reduction in Wnt/β-catenin signaling [119, 128, 129]. Dedicator of cytokinesis 4 (DOCK4) is another gene that plays an important role in Wnt signaling [123]. DOCK4 is a part of the destruction complex and its decreased expression is found to reduce the transcriptional activity induced by Wnt signaling [123]. In autism, levels of DOCK4 are found to be diminished, which leads to reduced Wnt signaling and the growth of dendrites [123, 130]. Several genes belonging to the non-canonical Wnt signaling pathway are found to be associated with autism [119]. The Prickle2 (Pk2) gene interacts with post synaptic density 95 (PSD-95). Mouse models with Prickle2 mutations displayed altered social, learning, and behavioral abnormalities, in addition to reductions in dendrite branching. Mutations in Ankyrin-3 (ANK3) and Prostaglandin E2 (PGE2) genes are associated with ASD [131–133]. ANK3 and PGE2 genes are important in neuronal development. PGE2 is regulated by Cyclooxygenase-2 (COX2) and is the main regulator of PGE2 synthesis. Studies have shown there is an association of abnormal COX2/PGE2 signaling with ASD [133]. All the findings suggest that both activation and inhibition of Wnt signaling pathway are associated with autism risk.

## Presynaptic Wnt signaling

Canonical Wnt signaling or Wnt/β-catenin signaling plays an important role in the development of synapsis in the pre- and post-synaptic terminals. The release of neurotransmitters at the presynaptic terminal is triggered by the binding of Wnt ligand to a receptor that activates Dishevelled-1 (DVL1). This binds to the pre-synaptic proteins, such as Synapsin-1 (SYN1) and Synaptotagmin (SYT), that are associated with ASD and enhance the clustering of synaptic vesicles (SV’s) and release of neurotransmitters. On the other hand, cell adhesion proteins, such as cadherins and cell adhesion complexes (NLGN/NRXN), play an important role in the modulation of presynaptic terminal activity. Their interaction regulates both excitatory and inhibitory synaptic function, disrupting the E/I balance in the postsynaptic neurons. β-catenin is bound to cadherins and their interaction is essential for the recruitment of SVs to synapses. MET, a receptor for hepatocyte growth factor (HGF), is found to have a genetic link with ASD. MET phosphorylates Tyr-142 of β-catenin and promotes its dissociation from the cadherins leading to its release in the postsynaptic terminal [119] (Figure 4). Wnt/β-catenin signaling plays a significant role in ASD as it is involved in stabilizing the synaptic structure by enhancing clustering of SVs, release of neurotransmitters and modulation of cell adhesion complexes [134].

## Postsynaptic Wnt signaling

Activation of the Wnt/β-catenin signaling causes Wnt ligands to bind to the Frizzled-9 (FZD9) receptor that recruits the multi-protein destruction complex consisting of scaffolding proteins (APC and Axin), phosphatases (PP1 and PP2A), and kinases (GSK3β and CK1). This activation inhibits GSK3β and prevents the degradation of β-catenin, thus stabilizing it and translocating it to the nucleus, leading to gene transcription [123] (Figure 4). Conversely, canonical and non-canonical Wnt signaling are associated with Ca^2+^ signaling. Wnt ligands are found to increase the influx of Ca^2+^ in neurons, and voltage gated Ca^2+^ sensitive channels, like NMDAR, allows the entrance of Ca^2+^ in the postsynaptic membrane that allows long term synaptic potentiation (LTP) establishment. Both high and low responses of LTP are associated with ASD. Ca^2+^ causes the activation of CAMKII, which is involved in the reorganization of cytoskeleton, calcineurin (CaN), a calcium dependent protein phosphatase and protein kinase C (PKC). The activation of CAMKII leads to the activation of transcription factor CREB, causing its translocation to the nucleus, which leads to gene transcription [135].

## Hyperactive pro-growth signaling pathways involved in ASD

### mTOR

mTOR, a highly conserved serine/threonine kinase, is important in the regulation of cell growth, cell metabolism and cell survival processes [136]. The catalytic subunits of mTOR consist of two structurally and functionally distinct protein complexes known as mTORC1 and mTORC2, which are involved in integrating information in the brain received from various intracellular and extracellular responses [137]. As mTOR is shown to be a major role player in the regulation of various processes that control synthesis of proteins, dynamics of actin cytoskeletal, regulating energy homeostasis and metabolism, the dysregulation of these pathways can lead to disorders including ASD, and neurodegeneration [138–140]. The disruption of mTOR pathway is associated with ASD and several other disorders that are caused by genetic alterations in mTOR pathway members, such as TSC1, TSC2, and PTEN. Also, mTOR activity levels can serve as indicators of disrupted growth states in the brain [141]. Studies have shown that several mTOR substrates such as p70 ribosomal S6 kinase 1 (S6K1) and the eukaryotic translation initiation factor 4E-binding proteins (4E-BPs) are found to be associated with ASD [142]. In particular, deletion of 4E-BP2, which is also a downstream effector of mTOR results in elevated dendritic spine density, and various behavioral abnormalities that are reminiscent of ASD [143–146]. mTORC1 complex promotes the ribosome production and translation by phosphorylating S6K1 through various effectors [147]. Hyperactivation of mTORC1 is responsible for ASD symptoms as one study has showed that mTORC1 hyperactivation in cerebellar Purkinje cells resulted in autistic like behaviors in mice [148] and it can be targeted for the treatment of ASD by modulating its expression.

### Brain- derived neurotrophic factor (BDNF)

Neurotrophins play an important role in the nervous system and are involved in the regulation of neuronal development, survival, morphogenesis, differentiation and synaptic plasticity [141, 149]. The most abundant neurotrophin that is found in the central nervous system (CNS) is the brain- derived neurotrophic factor (BDNF) [149]. The control of neurotrophin signaling at different epigenetic, transcriptional and translational levels are critical for the overall connectivity of neurons and other physiological functions, such as cell fate, axon and dendritic growth and synaptic pruning [150, 151]. The dysregulation of neurotrophin signaling has been reported in various neurological disorders, such as Alzheimer’s, Huntington’s disease and autism [152–154]. Under normal conditions, receptors such as FMRP, TSC1/2, and PTEN regulate the excitatory activity induced by BDNF by acting on its receptor TrkB. However, in pathological conditions such as ASD, lack of this regulation leads to disruption of synaptic functions [155]. The pro-growth signaling pathways induced by other trophic factors such as insulin-like growth factor (IGF), vascular endothelial growth factor (VEGF), glial-derived neurotrophic factor (GDNF) and ciliary neurotrophic factor (CNTF) are found to be dysregulated in ASD and used to categorize ASD into neural overgrowth or undergrowth types [141].

### ERK/MAPK

ERK1/2 are members of the MAPK signaling cascade and play important role in the regulation of cell growth, proliferation, differentiation and apoptosis [156]. MAPK/ERK pathway is involved in the signal transduction from cell surface receptors to the nucleus and they respond to growth factors, oxidative stress, chemokines and cytokines [157]. The activation of ERK1/2 is essential for dendritic spines formation and stabilization [158, 159] as well as supports the development of cerebral cortex by regulating cell cycle in neural progenitor cells proliferation. It is observed that the ERK/MAPK signaling interacts with many genes and CNVs implicated in ASD [160]. Activated or blocked levels of phospho-ERK1/2 are found to be associated with autistic phenotypes [161, 162]. Thus, targeting the ERK/MAPK pathway can be used to treat cognitive and behavioral impairments implicated in ASD [160] and a recent study performed in a mouse model with 16p11.2 deletion, which is a CNV associated with autism, showed that treatment with ERK inhibitor during period of development rescued anatomical and behavioral deficits in the mice [163].

## Other signaling pathways involved in ASD

There are many ASD genes that have been associated with different signaling pathways and that contribute to different ASD phenotypes (Table 1). One such pathway is the Calcium (Ca2+) and calmodulin (CaM) signaling pathway. These pathways play a significant role in the connectivity of neurons and functioning of the synapse, so dysregulation of this pathway might be responsible for the development of ASD [164, 165]. Impaired Ca2+ signaling has been found in many ASD individuals [166–168]. In skin fibroblasts derived from ASD individuals, the agonist evoked Ca2+ signaling was found to be dysfunctional [167]. Ca2+ signaling dysregulation and activity-dependent gene transcription changes were reported in a study involving induced Pluripotent Stem Cells (iPSC) that were derived from ASD individuals diagnosed with Timothy syndrome [169]. A study identified the common variants of ASD risk genes that regulated FMRP signaling showed that a SNP in the calcium/calmodulin-dependent kinase IV (CaMKIV) gene, which is a positive regulator of the FMRP transcription, causes a higher risk for the development of ASD [170]. Similarly, a *de novo* mutation of Glu183 to Val (E183V) in the catalytic domain of CAMKIIα increased dendritic branching and decreased synaptic transmission with reduced dendritic spine density causing ASD-related behaviors in mice [171].

**Table 1:** Studies showing ASD associated genes that contribute to ASD phenotypes through different signaling pathways.

**Genes in ASD**	**Genes affecting signaling pathways**	**Mutations contributing to autistic phenotypes**	**References**
Calcium/Calmodulin Dependent Protein Kinase IV (CAMKIV)	CaM signaling	Deficits in learning and memory formation	[13, 119]
Calcium/Calmodulin Dependent Protein Kinase II (CAMKIIα)	CaM signaling	Memory impairment	[13, 171, 220]
Synaptic Ras GTPase Activating Protein 1 (SYNGAP1)	Excitatory/glutamatergic signaling	Non-syndromic mental retardation	[42, 221]
Glutamate Ionotropic Receptor NMDA Type Subunit 2B (GRIN2B)	Excitatory/glutamatergic signaling	Deficits in learning and memory	[42, 222]
Fibroblast Growth Factor 7 (FGF7)	FGF signaling	Epileptic seizures	[116, 184]
Metabotropic glutamate receptor (mGLUR5)	FGF signaling	Aberrant dendrite growth leading to cognitive abnormalities	[119, 223, 224]
Sodium Voltage-Gated Channel Alpha Subunit 1 (SCN1A)	GABA signaling	Cognitive and behavioral deficits	[225, 236]
Methyl-CpG Binding Protein 2 (MECP2)	GABA signaling	Cognitive and behavioral deficits, impaired coordination	[194]
Solute Carrier Family 6 Member 11 (SLC6A11)	GABA signaling	Cognitive deficits	[30, 227]
Neurexin 1 (NRXN1)	GABA and glutamate signaling	Cognitive impairments, behavioral and learning deficits	[228–230]
Glutamate Ionotropic Receptor Delta Type Subunit 1 (GRID1)	Glutamate signaling	Impaired emotional and social behaviors	[231]
Calcium Voltage-Gated Channel Subunit Alpha1 C (CACNA1C)	Glutamate signaling	Impaired memory, hippocampal plasticity and anxiety-related behavior	[232]
SH3 And Multiple Ankyrin Repeat Domains 1 (SHANK1)	Glutamate signaling	Increased anxiety, reduced long-term memory	[31]
SH3 And Multiple Ankyrin Repeat Domains 2 (SHANK2)	Glutamate signaling	Increased anxiety, impaired social behaviors	[31]
Glycine Receptor Alpha 2 (GLRA2)	Glycinergic signaling	Deficits in learning and memory	[102]
Tuberous Sclerosis Complex Subunit 1 and 2 (TSC1 and TSC2)	mTOR signaling pathway	Learning deficit and impaired social behavior	[233]
Neurofibromin 1 (NF1)	mTOR signaling pathway	Learning and attention deficits	[233]
Fragile X Mental Retardation 1 (FMR1)	mTOR signaling pathway	Cognitive deficits, increased anxiety	[233]
Contactin Associated Protein Like 2 (CNTNAP2)	mTOR signaling	Impaired social and repetitive behaviors	[234, 235]
Phosphatase and Tensin Homolog (PTEN)	mTOR signaling pathway	ASD like social behavior	[233]
Homer Homolog 1 HOMER1	mGLUR signaling	Learning and memory deficits	[60, 236]
Molybdenum Cofactor Sulfurase (MOCOS)	Purine metabolism pathway	Autistic features	[54]
Retinoid-Related Orphan Receptor-Alpha (RORA)	Retinoic acid (RA) signaling	Language impairment	[80, 237]
Forkhead Box N1 (FOXN1)	Retinoic acid (RA) signaling	Brain alterations contributing to autistic features (hypothetical)	[238, 239]
Aldehyde Dehydrogenase 1 Family Member A3 (ALDH1A3)	Retinoic acid (RA) signaling	Autistic traits	[183]
Patched Domain Containing 1 (PTCHD1)	Sonic hedgehog (SHH) signaling	Cognitive alterations	[116, 175]
7-Dehydrocholesterol Reductase (DHCR7)	Sonic hedgehog (SHH) signaling	Intellectual impairment	[116, 177]
Engrailed Homeobox 2 (EN2)	Sonic hedgehog (SHH) signaling	Deficits in social behavior	[116, 240]
Distal-Less Homeobox (DLX)	TGF-β/BMP signaling	Autism like behaviors	[116, 241, 242]
Thyroid Hormone Receptor Alpha 1 (THRA1)	Thyroid pathway	Impaired memory, anxiety, locomotor dysfunction	[243, 244]
Parkinsonism Associated Deglycase 2 (PARK2)	Ubiquitin pathway	Impaired speech and stereotypical behaviors	[89, 245]
Chromodomain Helicase DNA Binding Protein 8 (CHD8)	Wnt signaling (canonical)	Defective neural progenitor proliferation and differentiation	[121]
Catenin Beta 1 (CTNNB1)	Wnt signaling (canonical)	Defect in brain development	[246, 247]
Prickle Planar Cell Polarity Protein 2 (PRICKLE2)	Wnt signaling (non-canonical)	Abnormalities in behavior, learning and social interaction	[248]
Transducin Beta Like 1 X-Linked (TBL1X)	Wnt signaling	Intellectual disability and autistic features	[249]
SH3 And Multiple Ankyrin Repeat Domains 3 (SHANK3)	Wnt signaling	Delayed or absent speech, intellectual disability	[58, 246, 250]
Adenomatosis Polyposis Coli (APC)	Wnt signaling	Memory impairment, autistic behaviors	[251]
Ubiquitin Protein Ligase E3A (UBE3A)	Wnt signaling	Developmental delay, learning difficulties	[252, 253]
Glycogen Synthase Kinase 3 Beta (GSK3β)	Wnt signaling	Anxiety and impaired social interaction	[13, 254]
Disrupted in Schizophrenia 1 (DISC1)	Wnt signaling	Failure is establishment of long-term synaptic potentiation (LTP) causing learning and memory deficits	[119]
Dedicator of Cytokinesis 4 (DOCK4)	Wnt signaling	Suppression of dendrite growth causing impairments in cognitive and language abilities	[123]
Transcription Factor 7 Like 2 (TCF7L2)	Wnt signaling	Cognitive and sensorimotor impairments	[119, 255]
Neuroligin 3 and 4 (NLGN3 and NLGN4)	Wnt signaling	Failure in synapse formation resulting in impaired communication abilities	[97, 119]
Dual Specificity Tyrosine Phosphorylation Regulated Kinase 1A (DYRK1A)	Wnt signaling	Head size abnormalities	[134, 256]
Transducin Beta Like 1 X-Linked Receptor 1 (TBL1XR1)	Wnt signaling	Delayed language development	[257]
DIX Domain Containing 1 (DIXDC1)	Wnt signaling	Impaired social behavior and anxiety	[116, 126]
Ankyrin 3 (ANK3)	Wnt signaling	Autistic features	[116, 258]
Prostaglandin E2 (PGE2)	Wnt signaling	Hyperactivity, repetitive behaviors and anxiety	[116, 259]

Another signaling pathway known as Sonic hedgehog (SHH) plays a major role in the developmental processes of multicellular embryos [172]. It is also the main component involved in the regulation of neural patterning and polarity of the CNS [173]. Within the forebrain, hindbrain and spinal cord, SHH signaling paces up proliferation and axonal targeting [174]. The SHH pathway has also been implicated in ASD, and mutations have been observed in patched domain-containing 1 (PTCHD1). A deficiency of this gene in male mice caused synaptic dysfunction and abnormal neuronal excitations leading to hyperactivity and cognitive alterations [175, 176]. Mutations in genes 7-dehydrocholesterol reductase (DHCR7) [177] and engrailed2 (EN2) have also been associated with ASD [178, 179]. In addition, similar cerebellar morphological abnormalities were displayed by mouse variants of EN2 and autistic individuals [180].

Retinoic acid (RA), derived from vitamin A (retinol), is a lipophilic molecule that is essential for vertebrate development and acts as a ligand for retinoic acid receptors (RARs) and retinoid X receptors (RXRs) [181]. Vitamin A deficiency leads to a number of abnormalities and induces autistic-like behaviors in rats by suppressing the expression of CD38 in the hypothalamus of the offspring [182]. Retinoic acid-related orphan receptor alpha (RORA) variants have been found in ASD, and decreased protein expression and abnormal methylation have been found in the autistic brain [80]. RORA regulates FOXN1 and ALDH1A3, enzymes that synthesize Retinoic Acid (RA). These RA signaling genes have been found to be associated with ASD [183].

Another signaling that is found to be associated with ASD is the fibroblast growth factor (FGF) signaling [184]. FGF belongs to family of cell signaling proteins that play an important role in brain patterning and any dysregulation of FGF signaling can lead to various neurological disorders [185]. The pathological role of FGF signaling in ASD was displayed by a study that reported impairment of synapse formation in hippocampal neurons in mutant mice lacking FGF7 [184].

The TGF-β superfamily consists of TGF-β/activin and the bone morphogenetic protein (BMP)/growth that plays an important role in bone organogenesis [186]. BMPs are important in nervous system development and their signaling is dysregulated in ASD. BMPs are involved in the activation of downstream Smads proteins and also interact with other signaling pathways such as MAPK, mTOR, Notch, Hedgehog and Wnt [186]. Distal-less homeobox (DLX) genes encoding homeodomain transcription factors are found to be dysregulated in ASD that results in alteration of BMP signaling [187, 188]. DLX genes are involved in craniofacial patterning and survival of inhibitory neurons located in the forebrain [187]. One of the genes, DLX5 was found to be overexpressed in a cell line with upregulation of the BMP-binding endothelial regulator (Bmper) [189].

A common pathophysiological mechanism that is disrupted in ASD is the imbalance in excitatory and inhibitory neurotransmission. The excitatory mechanism is mediated by glutamate, while the inhibitory mechanism is mediated by GABA [190, 191]. Studies have reported abnormalities in glutamate and GABA receptors expression in the postmortem brains of individuals affected with ASD [192]. One of the genes affecting the GABA signaling is the MECP2 gene and a study showed that MECP2 transgenic mice displayed stereotypic behaviors, ataxia, motor dysfunction and seizures [193, 194].

## Clinical aspects

Clinical diagnosis of ASD is primarily based on the analysis of complex behavioral and functional changes in patients during their developmental process. Genetic tests such as comparative genomic hybridization and chromosomal microarray (CMA), and G-band karyotyping can be used for the early diagnosis of ASD. CMA has been shown to have higher clinical yield and higher resolution as compared to the G-band karyotyping [195, 196]. Karyotyping is used to detect chromosomal abnormalities such as translocations or small portions of chromosomes in different disorders [197]. G-banding also known as Giemsa banding is a staining technique that is used to differentiate the chromosomal arms [198]. In ASD, G-banding is considered useful as it can help in the detection of chromosomal abnormalities in individuals affected with ASD [199]. CMA test can detect gene duplications and deletions associated with ASD [200], and proves to be more efficient in analyzing different types of variations present in ASD.

Improvement of emotional, physical and behavioral symptoms are one of the main aspects in the pharmacological treatment of ASD [201]. To treat these symptoms underlying ASD, one of the main goals is the identification of genes and biomarkers. Next generation sequencing (NGS) is a complex emerging clinical practice that is opening a new way for the identification of ASD-causing genes that includes abnormal social interaction and communication [202]. NGS helps in the identification of rare alleles, defects of single gene and variations of gene function. It includes whole-genome sequencing (WGS) and whole-exome sequencing (WES) [203, 204].

A wide range of antiepileptic drugs (AEDs) are used for effective treatment of ASD. AEDs are psychotropic drugs that modulate electrochemical activity in the brain and can induce a positive or negative affect on mood and behavior [205]. One of the reasons of using AEDs in treating ASD is high incidence of epilepsy in most of the ASD affected individuals. Research has shown that AEDs treatment improved communication and behavior in ASD affected individuals with epileptic discharges [206]. Valproic acid, lamotrigine, levetiracetam and ethosuximide are most commonly used AEDs, and they are found to reduce seizures in ASD individuals [207]. Another AED, topiramate when combined with an antipsychotic drug risperidone reduced hyperactivity, irritability and stereotypical behaviors in ASD individuals [208]. Some AEDs such as sodium valproate may have negative effect on the developing fetus if the mother takes this drug during pregnancy. Sodium valproate can lead to an abnormal brain development leading to neurological disorders such as intellectual disability and autism [209, 210]. Also AEDs such as lamotrigine can enhance or have a mood leveling effect, while AEDs such as levetiracetam are associated with side effects in behavior such as aggression, anxiety or nervousness and hostility [211, 212]. There is a limitation of the efficacy of AEDs medications in individuals affected with ASD because AEDs show improvement in only specific type of behaviors such as hyperactivity, impulsivity, mood instability, repetitive behaviors and aggression [213].

Although there are no pharmacological treatments that are able to treat the core symptoms of ASD but there are psychotropic drugs that have similar effect as AEDs in alleviating the common symptoms of ASD such as irritability, hyperactivity, lack of focus, mood dysregulation, and social withdrawal [214].

Oxytocin is another pharmacological agent currently being used to treat core ASD symptoms [215]. Controlled trial studies showed the intravenous administration of oxytocin in ASD patients improved the symptoms in various domains including social behavior [216–218]. Apart from the few limitations of oxytocin such as dosage establishment, children safety and route of administration, it can be used as a potential therapeutic agent to treat ASD [105].

Further studies are required to relate the pharmacological treatment with the genomic changes in ASD for better treatment planning. We expect that in few years there will be enough genomic data that can be used for the pharmacological analysis of patients with ASD.

## CONCLUSIONS

The genetic architecture of ASD is heterogeneous and differs in every individual. The current identification of ASD is mostly based on observation of behaviors and the genetics that underlie ASD are still an active area of research. Nevertheless, advancements in the study of various molecular mechanisms encompassing the genetics of autism and the identification of many ASD risk genes have opened a new way to study the pathophysiology of ASD. We envisage that the identification of new biomarkers, risk genes and associated genetic pathways may help in the early diagnosis of ASD, and improvement in clinical and pharmacological treatments of the disorder.
